# Gender-Specific Interactions in a Visual Object Recognition Task in Persons with Opioid Use Disorder

**DOI:** 10.3390/biomedicines11092460

**Published:** 2023-09-05

**Authors:** JoAnn Petrie, Logan R. Kowallis, Sarah Kamhout, Kyle B. Bills, Daniel Adams, Donovan E. Fleming, Bruce L. Brown, Scott C. Steffensen

**Affiliations:** 1 Department of Psychology, Brigham Young University, Provo, UT 84602, USA; petriejoann@gmail.com (J.P.); kbbills@noordacom.org (K.B.B.);; 2 Department of Neuroscience, Noorda College of Osteopathic Medicine, Provo, UT 84606, USA; 3 PhotoPharmics, Inc., 947 So, 500 E, Suite 100, American Fork, UT 84003, USA

**Keywords:** electroencephalogram (EEG), substance use disorder (SUD), opioid use disorder (OUD), event-related potentials (ERP), visual attention, visual evoked potential (VEP), visual cognitive processing, alpha and beta brain oscillations, gender-specific differences

## Abstract

Opioid use disorder (OUD)-associated overdose deaths have reached epidemic proportions worldwide over the past two decades, with death rates for men reported at twice the rate for women. Using a controlled, cross-sectional, age-matched (18–56 y) design to better understand the cognitive neuroscience of OUD, we evaluated the electroencephalographic (EEG) responses of male and female participants with OUD vs. age- and gender-matched non-OUD controls during a simple visual object recognition Go/No-Go task. Overall, women had significantly slower reaction times (RTs) than men. In addition, EEG N200 and P300 event-related potential (ERP) amplitudes for non-OUD controls were significantly larger for men, while their latencies were significantly shorter than for women. However, while N200 and P300 amplitudes were not significantly affected by OUD for either men or women in this task, latencies were also affected differentially in men vs. women with OUD. Accordingly, for both N200 and P300, male OUD participants exhibited longer latencies while female OUD participants exhibited shorter ones than in non-OUD controls. Additionally, robust oscillations were found in all participants during a feedback message associated with performance in the task. Although alpha and beta power during the feedback message were significantly greater for men than women overall, both alpha and beta oscillations exhibited significantly lower power in all participants with OUD. Taken together, these findings suggest important gender by OUD differences in cognitive processing and reflection of performance in this simple visual task.

## 1. Introduction

Substance use disorders (SUDs) and addictions [1,2,3,4] are recognized as complex, chronically relapsing disorders of brain function and cognitive processing that are still not well understood and are commonly undertreated [3,5,6,7,8,9,10]. Ongoing research indicates that these neurobiological disorders often begin with recreational use that deteriorates into compulsive and self-destructive drug-seeking behaviors despite intentional and repeated efforts by the individual to stop [1,3,11,12,13,14]. Accordingly, the 2019 US Centers for Disease Control (CDC) and the Department of Substance Abuse and Mental Health Services Administration (SAMHSA) reported that nearly 20.4 million Americans 12 years and older struggled with some type of SUD, yet less than 20% received treatment [15]. Alarmingly, estimates also suggest that the annual economic societal costs for US citizen struggling with opioid use disorder (OUD) are between 78.5 and 504 billion USD when considering loss of future family income, and 2.8% of the GDP when considering health care costs, unemployment, and lost potential and productivity brought about by the premature death caused by OUD [12,15,16,17,18,19,20]. Such costs may be even greater in future years, given that the opioid epidemic especially affects adults of prime working age (25–54 years), and mortality rates from overdoses more than tripled between 1999 and 2018 [21]. In 2021, the National Institute of Drug Abuse (NIDA) reported that more than 106,000 people in the US had died that year from drug-involved overdoses, including illicit drugs and prescription opioids [22,23]. Most reported deaths involved fentanyl, a powerful synthetic opioid that is similar to morphine, but is 50 to 100 times more potent [22,23]. Young-adult men (ages 25–39; urban and rural alike) have been affected the most by this opioid overdose epidemic, with their death rate at least double that of women in the same age group [21,22]. This discrepancy in mortality may suggest that physiological and/or psychological sex differences differentially inform response to opiates and associated withdrawal.

For the purposes of this study, the terms “women” and/or “men” or “female” and/or “male” are used interchangeably and refer to the biological sex and/or gender that our participants identified with at the time of this study. Discussion and research on the influence of biological sex or gender-specific differences on neural substrates that may influence cognitive responses to alcohol and other drugs (AOD) of abuse [24,25,26], including opiate/opioids [27,28,29], has historically been limited. However, with it now known that there is such biological diversity in those at risk for substance abuse or predisposition for opioid tolerance, dependence, and lethal OUD, and knowing that opioid overdose deaths happen in men at twice the rate of women, there is a great need for improved research in how treatment best works in SUDs [21,22]. Such research can inform evidence-based and individualized addiction treatment plans, which recognize any biological sex or gender-specific differences in the effects of certain drugs for males as opposed to females, including differences observed in individuals with opiate-related SUDs [28,30,31,32,33,34,35,36,37,38,39,40,41,42,43,44,45,46,47,48,49,50].

We aimed to expand this research in OUDs by observing potential gender-specific neurobiological differences in the visual cognitive processing of a simple Go/No-Go visual object recognition task in men and women with and without opioid use. Non-invasive electroencephalographic (EEG) scalp recordings with the accompanying analysis of visual evoked potentials (VEPs) and event-related potentials (ERP) are well-established neuroimaging techniques for recording brain function [51,52,53,54,55,56,57,58,59,60,61,62,63,64,65,66,67,68,69,70,71,72,73,74]. There is also hope that any chronic cognitive wavelength deficits and/or changes found in long-term substance use and subsequent SUDs can be recovered with detoxification efforts and ongoing recovery maintenance by the individual [62,64,67,75,76,77,78,79,80,81,82,83,84,85]. However, despite many studies measuring and documenting the maladaptive effects of chronic alcohol use disorder (AUD) via P300 ERPs [84], there are still few studies that measure this for other SUDs, including OUD [85]. Age-matched participants with no history of OUD (non-OUD) were compared to recently detoxified individuals with diagnosed OUD who were still in the addictive state of OUD craving and withdrawal while receiving in-patient treatment [64,81,84,86,87]. While collecting the EEG data, we also questioned whether reaction times (RT) would be another useful variable with measurable gender-specific differences when using our techniques and protocols. We hypothesized that there would be gender-OUD interactions for ERPs in this simple visual task.

## 2. Materials and Methods

### 2.1. Participants

The current study was designed specifically to obtain EEG measures of gender-specific differences in OUD [1,3]. All non-OUD control participants were recruited from the local community, including Brigham Young University (BYU), via Institutional Review Board (IRB)-approved announcements, word of mouth, and/or flyers. All subjects with OUD were recruited with the approval and help of the trained and licensed staff of a high-intensity residential and detoxification treatment facility in Utah, USA, adhering to 42 CFR Part 2 law [18]. This approved treatment center was a state-licensed, evidence-based detoxification and addiction rehabilitation center for the treatment of persons with or without mental health disorders co-occurring with SUDs and addiction, including OUD. The facility was required to meet the very stringent confidentiality and established regulations of the 1996 Health Insurance Portability and Accountability Act (HIPAA) [18] and provide SUD care according to the most current published American Society of Addiction Medicine (ASAM) Patient Placement Criteria. All diagnoses of OUD were based on the American Psychiatric Association’s (APA) Diagnostic Statistical Manual (DSM)-IV-TR development criteria [1,2]. However, it was understood that for the purposes of this study, the OUD cohort were all in various stages of withdrawal from their OUD, with time from last opioid use being as short as 24 h and no longer than 30 days out. Subjects ranged in age from 18 to 56 years. Once pre-screening self-report questionnaires were completed to ensure their basic good health, all participants from both cohorts were matched as closely as possible for age and gender [88] to yield the same mean age per group [84,88,89,90,91,92,93]: (1) Men with OUD (age range = 18–53 years); (2) women with OUD (age range = 18–51 years); (3) men with no-OUD as controls (age range = 17–53 years); and, (4) women with no-OUD as controls (age range = 18–56 years). After all EEG data were collected, only those data considered to be good quality were used in statistical analyses, as determined is best practice by at least 3 authors (see [87,94,95]). A final study sample size of 38 participants’ data was used in the four groups for this project: (1) Men with OUD (n = 10, age range = 20–53 years, M = 30, SD = 9); (2) women with OUD (n = 10, age range = 18–51 years, M = 30, SD = 12); (3) male controls with non-OUD (n = 9; age range = 21–46 years, M = 26, SD = 7); and (4) female controls (n = 9, age range = 18–56 years, M = 25, SD = 12). The mean age difference between participants with OUD and the non-OUD controls was four years.

### 2.2. Ethical Considerations

Strict adherence to ethical issues and research standards was maintained. All study procedures were approved by the Brigham Young University (BYU) Institutional Review Board (IRB) before recruitment started. Confidentiality and compliance met the 1996 Health Insurance Portability and Accountability Act (HIPAA) requirements for research data collected, and all clinical and research personnel were trained in confidentiality protocols and maintained the required Collaborative Institutional Training Initiative (CITI) certifications [96]. All personnel involved in the research project signed confidentiality agreements. Signed consent forms were also obtained from all participants prior to any testing. Subjects were then immediately given research identification numbers so their data could be anonymized. All electronic data were stripped of any unique identifiers (e.g., name, date of birth) and stored in a secure electronic database. No personally identifying information or data were entered into the multi-lab computer database or EEG console. All data entries were verified by two raters for accuracy.

### 2.3. Inclusion Criteria

Eligibility for each volunteer participant was initially established through a brief in-person interview with either a licensed staff member and/or a therapist at the recruiting treatment center or by a trained research assistant referring to an already established and approved Pre-Screening Checklist developed for the study. Participants with OUD were diagnosed following the DSM-IV-TR manual, were between ages 18–56 years, were detoxified from all substances including opioids but still experiencing severe daily craving for drugs, and had not used opioids in the past 24 h but were no longer than 30 days from opioid use. Inclusion criteria for non-OUD control participants required that there was no history of OUD or use of any opioid painkiller medication in the last three months or sleep aids in the past month, including no use of prescription painkillers or other mood- or mind-altering prescription drugs. Additional participation criteria for both groups were: male or female, age 18–56 years, no prior history of severe brain injury, seizure free, not prone to fainting, not claustrophobic, and willing to indicate all drug use on drug history sheet. Further, to ensure no current drug use confounding factors, a sample for urine analysis (UA) was required from all participants onsite at the time of testing. All UA samples were tested for a variety of drugs including opiates, marijuana/THC, cocaine, amphetamines, and benzodiazepines. No positive reports from the subsequent independent UA were obtained, and thus no participant data were excluded due to confounding substance exposure.

### 2.4. EEG Procedures

The current study is part of a 2 h assessment period session designed for comparing men and women with or without OUD in a controlled setting. This controlled session included a battery of standardized computerized and pencil-and-paper neuropsychological tests for assessment of cognitive processing capabilities and short-term memory tasks, and computerized paradigms coupled with EEG recordings. To limit EEG testing confounds as much as possible, all participants were tested for handedness and eye dominance, binocular rivalry, color vision deficiencies, and finger-tapping ability (for reaction time (RT) data collection). All participants also completed an online demographics/screening questionnaire prior to the EEG session to verify that they met the necessary study inclusion criteria. If all pre-qualification criteria were met, participants were scheduled immediately for their EEG session. To ensure that every participant was truly drug-free at the time of the study, rather than relying on self-report only, each participant provided a required urine sample for analysis (UA) before any testing continued. Each UA sample was immediately coded with their study ID number only and placed in a locked freezer in the lab until it could be transferred by the PI (J.P.)to the partner treatment center’s lab to be later tested for a variety of illicit drugs including opiates, marijuana/THC, cocaine, amphetamines, benzodiazepines, and phencyclidine (PCP). A positive UA would later indicate a need to discard participant data due to drugs in the system; there were no positive UAs found. All participants were again advised of their confidentiality rights and following consent. All other tests for the larger study were taken at the same time the UA sample was given [97].

### 2.5. Preparation for Evoked Potential Recording

Each participant’s head circumference was measured to determine the correct size EEG sensor net. To ensure a good cap fit, three different sizes of the 128-channel Geodesic EEG System™ (GES) HydroCel Geodesic Sensor Nets (HCGSN; Electrical Geodesics, Inc., Eugene, Oregon) were utilized in testing. The 10–20 placement system of the sensor net’s 128 electrodes was used for referencing points of the Average-Reference Mastoid Montage [98]. E-prime 3.0 software ^®^ (Psychology Software Tools, Inc., Sharpsburg, PA, USA) was used for the computerized visual attention tasks with EEG data transmission to a closed-circuit computer monitor screen placed directly in front (68 cm) of the participant. All collected EEG data were sent to an observation screen being monitored outside of the sound-proofed, dimly lit, and electromagnetic-shielded EEG recording room.

### 2.6. Object Recognition Task

**Figure 1:**
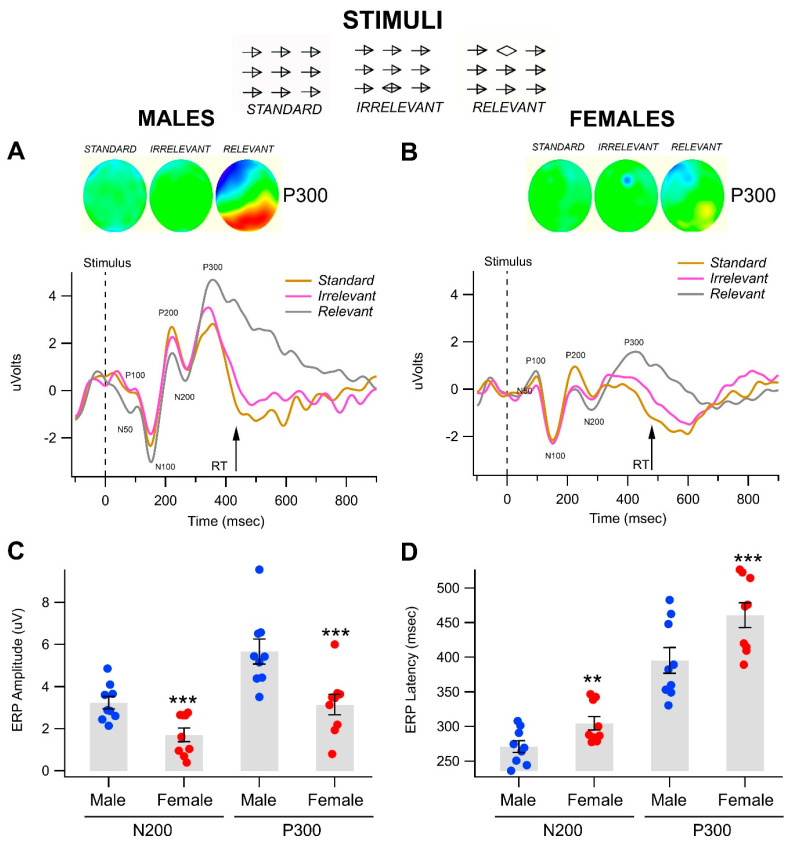
Gender-specific differences in event-related potentials in a visual object recognition task. Insets show the Standard, Irrelevant, and Relevant stimuli (50 ms) that were randomly presented at 2–4 s intervals over 10 min. Participants responded to the Relevant oddball open diamond “pop out” stimuli. (**A**,**B**) Each graph depicts superimposed grand-average visual event potentials (VEPs; N50, P100, N100, P200, N200, and P300) in response to Standard, Irrelevant, and Relevant stimuli during the Go/No-Go visual object recognition tasks. VEPs were recorded at electrode Pz in males (**A**) and females (**B**). Time of stimulus presentation is noted by dashed lines. Average Go Response reaction time (RT) is also noted by arrows. Of particular note is the stimulus-specific difference in P300 ERPs for the Relevant condition (vs. Irrelevant and Standard) in both men and women. The 128 sensor topo maps (colored circles) represent grand-averaged potentials obtained from across the head for all men compared to all women participants at 349 ms (P300) after the presentation of the stimuli. These maps are oriented as if looking down on the head from above (top of circle = front of head, bottom of circle = back of head). Extreme negative potentials are depicted in violet, and extreme positive potentials are shown in red. Again, note the differentiation across stimuli conditions of the P300 ERPs associated with the Relevant stimulus in both men and women on the topo maps and their prominence in the back of the head for the men but not for women. (**C**,**D**) These composite plots give a summary of descriptive statistics for ERP amplitude and latency measurements taken at location Pz for Relevant stimuli only across back of the head electrodes. They show the data points for each of the 38 participants by gender and ERP component. Male non-OUD subjects were characterized by significantly larger N200 and P300 amplitudes and shorter latencies than female non-OUD subjects. Note ** *p* < 0.001; *** *p* < 0.0001.

For this visual evoked potential (VEP) task, one object in the Relevant matrix of right-pointing arrows was an oddball “open” diamond symbol and one object in the Irrelevant matrix was a variation of a diamond and arrow. The Standard matrix had only right-facing arrows (see Figure 1 for example matrix). Before beginning the object recognition task, each participant was shown a sample visual stimulus on the computer screen and instructed to respond with a simple key press whenever the oddball “open” diamond was observed in the Relevant matrix. Visual evoked potentials (VEPs) were acquired in 2 s epochs 100 ms before the stimulus and 1900 ms after the stimulus for each visual presentation, with the three 3 × 3 matrices randomly presented at 2–4 s intervals during the 8 min recording session. In order to avoid errors of the gaze throughout the trial, the targeted symbols for each matrix were also randomly positioned on the computer screen. Regardless of the matrix presented, these oddball elements were readily distinguished by participants as a “pop-out” from the other eight elements of each matrix, even with very brief presentation to the participant (50 ms). Reaction times (RT) were measured from the time the stimulus was presented until the participant pressed the button. If the participant correctly detected the oddball target, the word “Correct” was displayed as feedback on the computer screen 500 ms after their reaction time, with their reaction time posted. When no target was presented and participants did not press a button, the words “No response detected” were shown on the screen at 900 ms after the stimulus presentation. An “Incorrect response” notice appeared if the participant inadvertently pressed the button when no target object was in the matrix.

### 2.7. Wavelet Analysis

Alpha and beta brain oscillation power values were also calculated by comparing individual frequency bands with the continuous wavelet analysis tool in Net Station 4.5.7^®^ (Electrical Geodesics, Inc., 2013 (Computer Software), Eugene, OR, USA). A wavelet is a waveform similar to sine waves; however, it terminates at zero at each end. This allows the wavelet data analysis tool to decompose the waveform and calculate a power statistic at each time point for each wave’s frequency band of interest. Net Station 4.5.7^®^ uses a Morlet wavelet, which is a symmetrical waveform created by multiplying a Gaussian envelope by a sine wave. Whenever a waveform is analyzed, there is a tradeoff between accuracy in the frequency scale and accuracy in the time scale. We used the default settings for the Net Station 4.5.7^®^ wavelet tool (1.95–30.27 Hz, frequency step = 0.98, frequency scale factor = 5, window type = Cosinus, taper length = 0.10 s). Pre-processing involved 1–60 Hz bandpass filter, then segmentation (100 ms pre-stimulus to 1800 ms post-stimulus for a total of 1900 ms per segment), and finally baseline correction (100 ms pre-stimulus, 100 ms duration). The sampling rate was 250 Hz, so each 1900 ms segment (100 ms pre- + 1800 ms post-stimulus) included 475 time points. Therefore, the continuous wavelet analysis included 475 wavelet coefficients per segment. We exported the wavelet power coefficients to text files, organized and labeled the data using R (version 3.6.3) [99], and calculated statistics using SAS^®^ (version 9.2) [100].

### 2.8. Statistical Analyses

As observed ERPs and feedback oscillations were most evident in the back of the head, the analysis was simplified to include only seven occipital electrodes: Oz, Pz, P1, P2, O1, O2, and POz. Amplitude and latency were measured for the within-participant average VEP components for the N100, P100, N200, P200, and P300 using Net Station 4.5.7^®^ Data Analysis Tools (Electrical Geodesics, Inc., 2013 (Computer Software), Eugene, OR, USA). The quantitative electrophysiological data obtained through the EGI Data Analysis Tools^®^ were analyzed using the SAS (r) Proprietary Software 9.4 (TS1M5) and its mixed model SAS/STAT^®^ Proc Mixed 14.3 statistical analytics software program (SAS Institute Inc., Cary, NC, USA). This particular statistical program was developed to fit multilevel and hierarchical linear models and considered suitable for analyses of the “mixed” statistical data from the current study where data on individuals were nested within naturally occurring hierarchies such as men and women within the OUD cohort. A double-multivariate analysis of variance (MANOVA) approach was used to account for the several dependent variables (DVs) of the current study. This approach also used a typical MANOVA design for calculating a first set of independent variables (IVs), followed by a higher hierarchical analysis for a second set of IVs. The first set of IVs for our design included gender, OUD status, and gender-by-OUD status, nested within the second set of IVs: component, gender, and condition. Component included types of measurement, such as N200 and P300. Condition included different stimulus conditions: Standard, Irrelevant, and Relevant. In addition, Igor Pro 8.1^®^ software (WaveMetrics, Lake Oswego, OR, USA) was used to present the data in graphic form, while measures of reaction times (RT) were analyzed using one-way analysis of variance (ANOVA).

## 3. Results

### 3.1. Gender-Related Differences in Event-Related Potentials in the Visual Object Recognition Task

Throughout the Go/No-Go object recognition task’s recording session, matrices of visual stimuli (see Standard, Irrelevant, and Relevant examples in Figure 1) were randomly presented at 2–4-s intervals for 50 ms durations. Figure 1 compares grand-averaged visual evoked potentials (VEPs) elicited by Standard, Irrelevant, and Relevant stimuli at the Pz electrode for both men (Figure 1A) and women overall (Figure 1B) (n = 19 for each group). Parietal and occipital electrode sites provided the most well-defined combination of early (i.e., task-independent) and late (task-dependent) components of the VEP, and ERPs were generated in this Go/No-Go task, as shown in the topo maps for each of the stimuli in both men and women participants (Figure 1A,B).

Statistical analyses showed no significant differences in the N100, P100, or the P200 between men and women. However, in line with previous studies on substance use and abuse, N200 and P300 amplitudes within the grand-averaged wave forms were more strongly associated with Relevant stimuli conditions than with Irrelevant or Standard conditions. This trend was especially notable in occipital and parietal areas. Stimuli-specific changes in such amplitudes can suggest that this simple visual task was able to effectively differentiate between cognitive states. Overall, women had significantly slower RTs compared to men (men: 434.2 ± 6.9 ms; women: 480.6 ± 11.5 ms; F(1,37) = 10.3, *p* = 0.003; n = 19 each; see Figure 1A,B). Thus, this simple visual oddball or pop-out object recognition task evinced robust differentiation of cognitive potentials, despite individual variations in latencies of N200 and P300 and RT across participants. While the grand-averaged VEPs and topo maps demonstrated differences between responses obtained with the Relevant stimulus, it should be noted that averaging waveforms often underestimates the significance of the effects because of many factors including temporal dispersion, differences in latencies between subjects, and so forth. Therefore, in an effort to ameliorate issues involving temporal dispersion and massive grand averaging, we measured components of the VEP in each participant separately with windowing and adaptive mean procedures and obtained individual measurements of amplitude and latency. (See Figure 1C,D, the distribution of amplitude and latency measurements for N200 and P300 for the Relevant stimulus at the posterior Pz electrode or all men vs. women participants as a representative example.) MANOVA revealed a significant overall effect of gender (Table 1).

### 3.2. Effects of Opioid Use Disorder on Event-Related Potentials in a Visual Object Recognition Task

**Figure 2:**
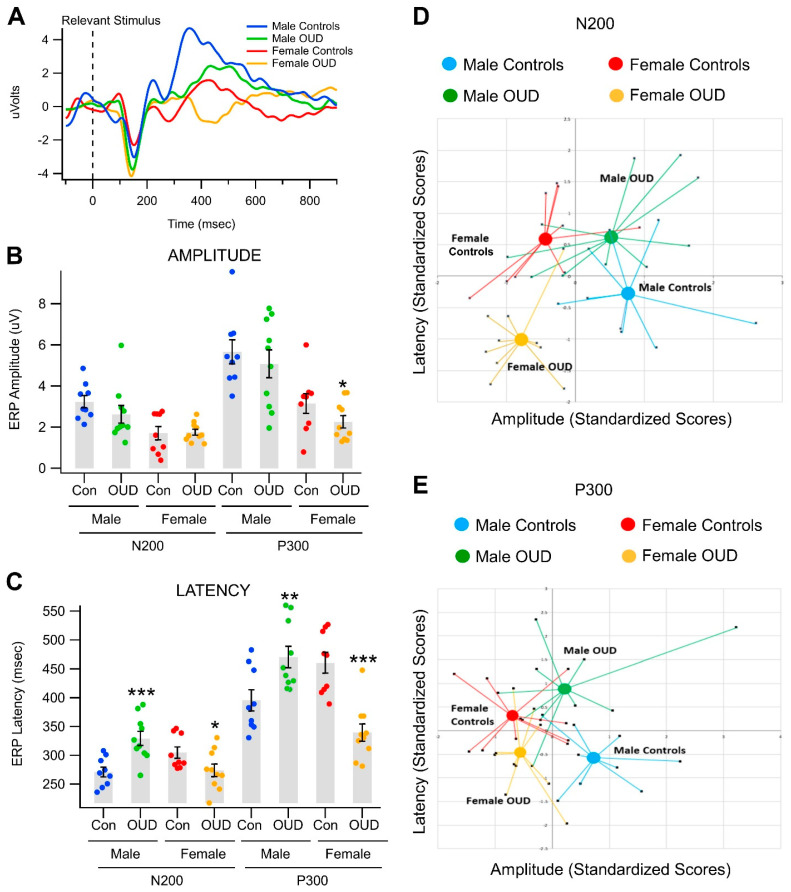
Effects of opioid use disorder on event-related potentials in a visual object recognition task. (**A**) Grand averaged visual event potentials (VEPs) obtained at Pz in men and women non-OUD controls vs. men and women with OUD for the Relevant stimulus. Compared to controls, those with OUD were characterized by smaller P300s on the grand-averaged waveform. (**B**) This graph summarizes ERP amplitude measurements in men and women and those with OUD. Scatter plots represent all the values in each group. Men had significantly greater N200 and P300 amplitudes compared to women regardless of OUD. However, there was a significant decrease in P300 ERP amplitudes for women with OUD. (**C**) This graph summarizes ERP latency measurements in men and women and those with OUD. Men had significantly shorter N200 and P300 latencies compared to women. Most interestingly, those with OUD had significantly longer N200 and P300 latencies in men, but shorter latencies in women. (**D**,**E**) In order to better demonstrate this double dissociation, standardized values for all participants by latency vs. amplitude are plotted in vectors to show the differential contrast between OUD and gender for N200 (**D**) and P300 (**E**).Note * *p* < 0.05; ** *p* < 0.001; *** *p* < 0.0001.

We compared grand-averaged VEPs obtained in non-OUD controls vs. OUD participants in the object recognition task (Figure 2A,C; shown here for the Relevant stimulus). MANOVA revealed a significant overall effect of OUD (Table 1). OUD effects nested within gender were significant for both P300 latency (*p* < 0.0001, eta-squared = 0.462) and N200 latency (*p* = 0.0004, eta-squared = 0.356; mixed-model correction verified these results), but not amplitude (Table 2). The gender-by-addiction interaction becomes more evident in the bivariate scatterplots in Figure 2D,E. Therein, latencies and amplitudes are standardized and then plotted for each participant in the study. This demonstrates differential responsiveness in men vs. women for both the N200 and P300, with latency being the primary factor. Clear clusters emerged for the differential relationship of OUD and gender in all participants. However, we also found that, although men and women with OUD had longer RTs than the non-OUD controls (men with OUD: 468.2 ± 15.8 ms; women with OUD: 516.4 ± 23.9 ms), there were no significant RT differences between non-OUD male controls vs. those males with OUD (*p* > 0.05); or between the non-OUD female controls and those females with OUD (*p* > 0.05).

### 3.3. Gender-Related Differences in Alpha and Beta Oscillations in the Visual Object Recognition Task

**Figure 3:**
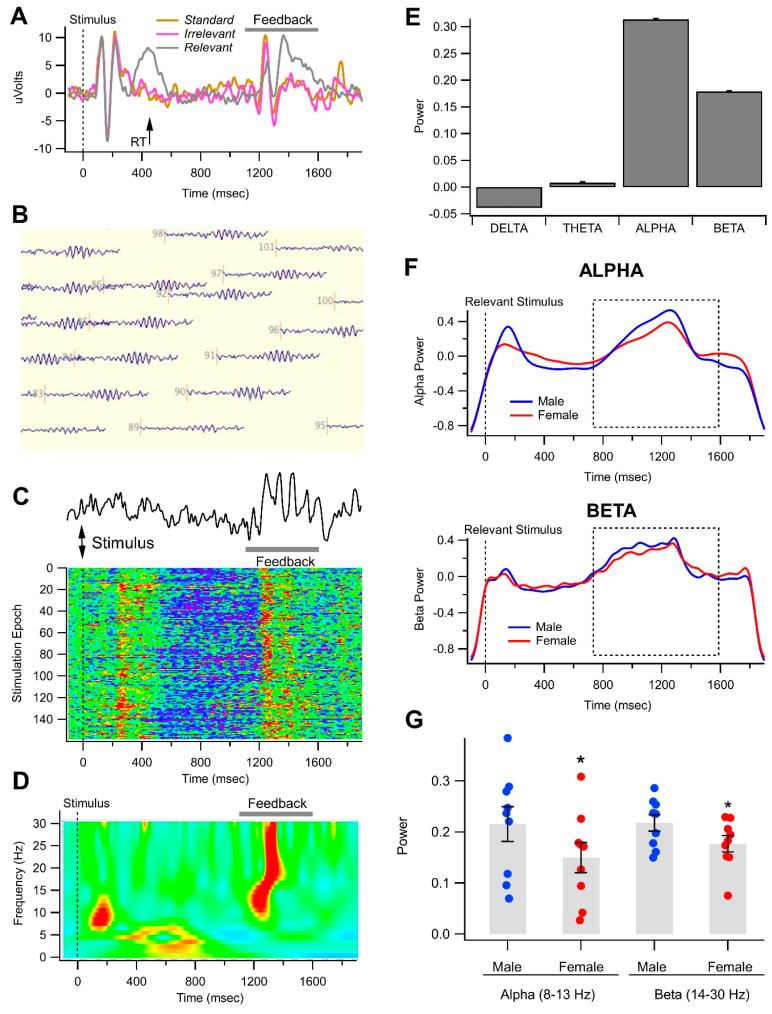
Gender differences in alpha and beta oscillations in a visual object recognition task. (**A**) This is a representative 2 s trace of raw electroencephalogram (EEG) data obtained in one non-OUD male participant at Pz for a Relevant stimulus. Note the feedback oscillations that occurred at around 1200 ms after the stimulus, when the feedback message was delivered to the subject. (**B**) Raw EEG data from a Relevant stimulus epoch in the same subject in (**A**) for all electrodes on the head. Top is front and bottom is back of the head. Note the oscillations that appeared during the feedback stimulus all around the head, but most evident at the back of the head. (**C**) Raw EEG epoch from one back of the head electrodes in (**B**) showing the robust oscillations during the feedback message as a Raster color plot of individual traces obtained for each of the 154 stimuli presented in one session for this representative participant. Red represents high positive amplitudes and violet represents low. Note that the oscillations are present for most of the epochs. (**D**) Wavelet analysis of a Relevant stimulus epoch from the same electrode shown in (**C**). Red indicates positive power while violet indicates negative. Note that in this subject there is robust theta, alpha, and beta power during the feedback stimulus. (**E**) This graph shows wavelet analysis of the average power for 4 bands during the feedback Relevant stimulus in this subject. Note that the feedback oscillation is dominated by alpha and beta activity. (**F**) Superimposed grand-averaged wavelets of alpha (8–13 Hz; above) and beta (13–30 Hz; below) activity in males vs. females for the Relevant stimuli. Note that alpha and beta activity is high around 1200 ms after the stimulus, which reflects the raw waveforms and wavelet analysis in (**B**–**D**). The dashed-line box indicates the window during the feedback stimulus which was used for determination of alpha and beta power. (**G**) This graph shows alpha and beta power (trapezoidal integration of area in dashed boxes shown in (**C**,**D**) over the epoch) for all men compared to all women for the Relevant stimulus condition across back of the head electrodes. Note that both alpha and beta power are significantly higher in men overall than for women Note * *p* < 0.05.

Upon inspection of visual evoked potential (VEP) waveforms, it became obvious that there was an evoked potential in some subjects associated with the feedback message that had components that mirrored those associated with those of the original VEP (Figure 3A). Figure 3A–E shows representative data from one male non-OUD control participant.

We observed high-power oscillations for most trials in this participant’s raw EEG recordings during the time interval when the 500 ms feedback message was presented. These feedback oscillations were most evident in electrodes at the back of the head (Figure 3B). The oscillation shown in Figure 3C is from one occipital electrode of this participant, and the oscillation persisted for the duration of the feedback message. However, there was no clear pattern of occurrence or amplitude of alpha/beta oscillations in the raw EEG by epochs of stimulus presentation (Figure 3C). Wavelet analysis in this subject revealed that the feedback oscillation was composed of mostly alpha and beta power (Figure 3D). Wavelet analysis also demonstrated that the oscillation consisted of theta, alpha, and beta frequencies during the time of the feedback message for one epoch of stimulation (Figure 3D,E). Figure 3E shows averaged wavelet power for the four frequency bands: delta (1–4 Hz), theta (4–8 Hz), alpha (8–13 Hz), and beta (13–30 Hz) for this male control participant evaluated from 700–1600 ms after the Relevant stimulus from an average of seven electrodes in the back of the head. Since more than 90% of the oscillation was dominated by alpha/beta power, we focused on these two bands for gender and OUD comparisons. Grand-averaged wavelet waveforms revealed slight differences in alpha and beta power between males and females (Figure 3F). Comparisons were then made between the alpha and beta wavelet power for all women vs. all men regardless of OUD during the feedback stimulus by using within-subject measurements rather than grand averaging waveforms. MANOVA revealed that there were significant differences between genders for both alpha and beta power, with females being lower in both alpha and beta power overall (Figure 3G; Table 1).

### 3.4. Effects of Opioid Use Disorder on Alpha/Beta Oscillations in a Visual Object Recognition Task

**Figure 4:**
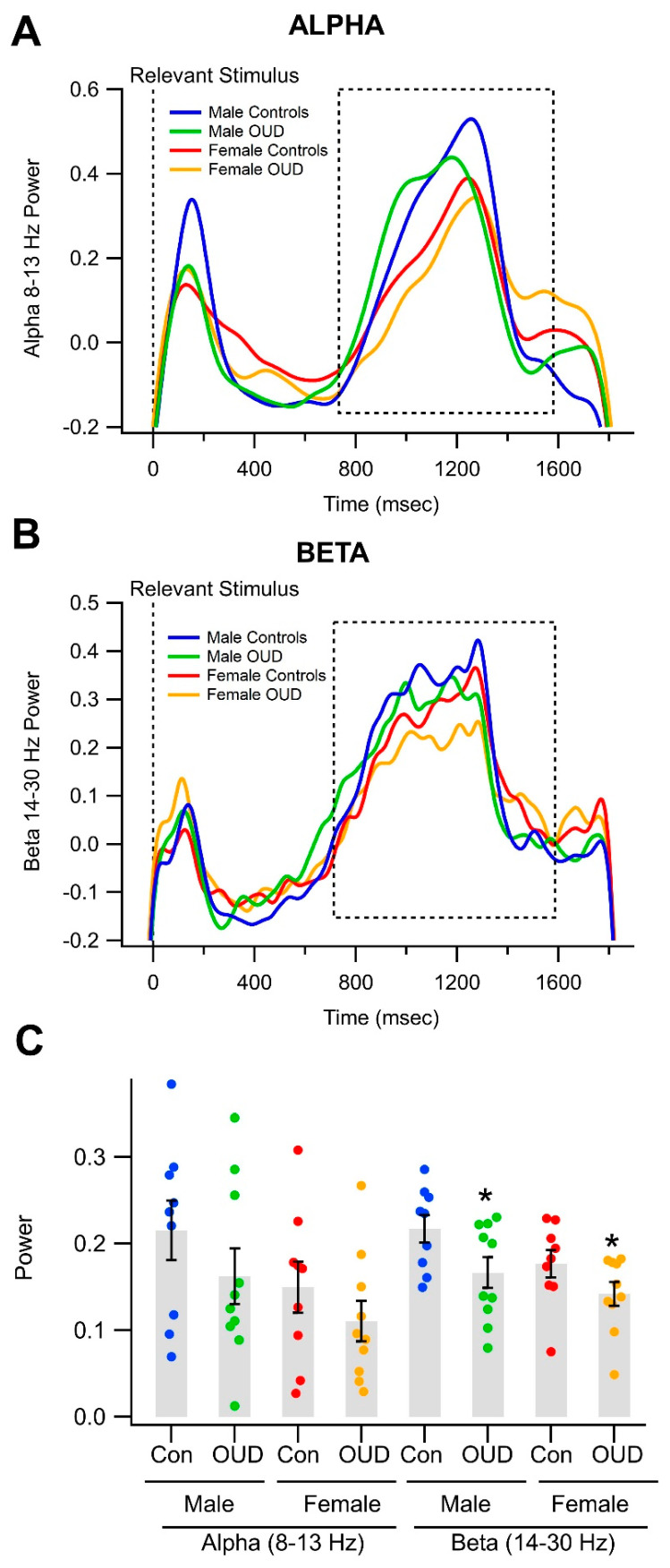
Effects of opioid use disorder (OUD) on alpha and beta oscillations in a visual object recognition task. (**A**) Superimposed grand-averaged alpha and beta (**B**) power determined with wavelet analysis comparing men vs. women and OUD vs. non-OUD participants. There appears to be a tendency for men’s waveforms to be greater than those for women during the feedback stimulus shown during the dashed-lined box corresponding to the feedback stimulus. (**C**) This graph shows alpha and beta power measurements for all men compared to all women and OUD vs. non-OUD. Although the trend was for OUD male and female subjects to have lower have lower alpha power, it was not significant. However, beta power was significantly lower in men and women participants with OUD vs. non-OUD controls. Note * *p* < 0.05.

We then compared alpha and beta power during the feedback stimulus in OUD vs. the non-OUD participants. Grand averages of wavelet waveforms during feedback oscillation revealed only minor differences across all OUD vs. all non-OUD participants for alpha and beta power (see Figure 4A,B). Figure 4C shows averages of alpha and beta power measurements for all participants in the current study.

Although there were no univariate differences in alpha power between OUD vs. non-OUD participants, there was a significant difference in beta power during the feedback stimulus between men with OUD vs. non-OUD men participants, with OUD men having less beta power (Figure 4C). Overall, OUD effects nested within gender were significant (*p* = 0.0333, η^2^ = 0.165). Roy’s Greatest Root—one of the four corresponding multivariate tests for this effect—is also statistically significant (*p* = 0.0261), hedging against the alpha inflation problem of multiple testing. Table 3 summarizes all correlations for all dependent variables (DVs) used in the MANOVA models. There was a strong correlation between N200 and P300 ERPs but only weak correlations with the other DVs like alpha and beta power.

## 4. Discussion

### 4.1. Event-Related Potential (ERP) Studies

Non-invasive electrophysiological scalp recordings in electroencephalograms (EEGs) have been recognized as objective means of observing neuronal information processing. Event-related potentials (ERPs) and visual event potentials (VEPs) are linked with normal and abnormal cognitions [101,102], including those associated with chronic drug use. For almost 3 decades, alternations in P300 ERPs in opiate and cocaine users have been shown to be meliorated by buprenorphine treatment [103], yet those findings did not amount to, or advocate for, improving the human condition of those individuals struggling with opioid addiction and OUDs.

Building on a previous EEG study [87], the current study investigates how persons with substance use disorders (SUDs) and addictions—in particular OUD—process a simple Go/No-Go visual object recognition task. As grand-averaged VEPs suggested potentially important gender differences in these data, we compared ERP latencies and amplitudes during the Go/No-Go visual object recognition task for male and female participants with and without OUD [30,64,75,103].

#### The P300 and N200 as Good Measures of OUD

Previous research has identified the P300 ERP as an objective measure of brain activity that responds to central nervous system (CNS) disruptions and pharmacologic manipulation [84] and may represent unique biological and/or genetic factors [53]. For over 40 years, decreased P300 amplitudes with no consistent differences in latencies have been identified in acute ethanol and marijuana use [64,104]. However, similar research has associated reduced amplitudes with increased latencies in chronic alcohol abuse [105,106] as well as within opioid addiction [52,84,103,107]. Reduced P300 amplitudes with no apparent changes in latency have also been observed in acute cocaine use [52,57,75,108]. Low P300 amplitude or P300 amplitude reduction is thought to be a strong indicator of the state of disinhibition [109,110,111,112] and is implicated in behaviors associated with early onset of SUDs [77,81], including the emotional disinhibition found in the alcohol-naïve children of those with alcohol use disorder (AUD) when performing a NoGo task [62]. In addition, the N200 ERP reflects a mismatch process associated with the orienting reflex [113]. Increases in the N200 latency have been related to increased effort in stimulus discrimination [114], while reduced N200 amplitude and longer latency have been found in those abstinent from AUD [79]. Increased N200 amplitude has also been observed in those with AUD, and these findings are similar to those associated with schizophrenia [115]. Because ERP measurements can quickly and affordably monitor electrical events which are associated with the development of drug tolerance and withdrawal, research on electrophysiological changes in visual processing may illuminate key neuronal mechanisms which may be modified in opiate addiction and OUD.

Our central aim was to use electrophysiological (EEG) scalp recordings to investigate gender-linked behavioral, physiological, or molecular markers in men and women with and without addiction and opiate use disorder (OUD). Our primary hypothesis was that there would be significant gender-by-OUD interactions in ERPs. Specifically, we wanted to observe N200 and P300 event-related potentials (ERPs) and brain function of those women and men struggling with OUD when compared to healthy non-OUD control participants by use of visual stimuli in a controlled setting. We also hypothesized that a basic neurophysiological marker might provide an objective index for the efficacy of treatment strategies for addiction—in particular opiate addiction and OUD. While N200 and P300 amplitudes were not significantly affected by OUD for either men or women in this study, latencies were significantly affected, and differentially, in men vs. women with OUD. Male OUD participants exhibited longer and female OUD participants exhibited shorter latencies, for both N200 and P300, than for non-OUD controls. These findings suggest that one can effectively use an EEG to objectively measure differences in ERPs over time and monitor improvement via simple individual EEG data studies. These techniques could be applied throughout initial treatment and beyond, and potentially help identify changes in biological patterns that could indicate risk of overlap in OUD, as we continue to face the question of why OUD overdose deaths happen in men at twice the rate as for women. Significant differences in patterns observed in males and females with opiate addiction suggests that the groups are distinct from one another. These findings are also in line with previous studies demonstrating reduced P300 amplitudes in males with alcohol and cocaine addiction, as compared with healthy male controls [33,34,35,52,79,114,116,117]. Until recently, little has been researched or said about females and addictions and SUDs [29], especially regarding P300. In addition, there are few ERP studies of visual cognitive processing involving P300s differences between men and women. The current study of object recognition stimuli in a Go/NoGo task was designed to address that gap, following two previous EEG studies that found P300s and N200s do not significantly vary across the menstrual phases, suggesting that the menses cycle does not account for possible cognitive processing differences between men and women [118,119].

### 4.2. Potential Limitations

Potential limitations of this ERP/EEG study may come from the smaller sample size of those with OUD being sorted to even smaller gender-specific cohorts. However, we did see distinctive variations in ERPs and oscillations that match other EEG studies of SUDs. Traditionally, very few of the EEG studies of SUDs of any kind have reported large sample sizes, which may be due to the societal stigmas that surround substance abuse and addictions [120,121]. Other confounds may come from the lack of EEG research specific to OUD, as polysubstance use and abuse is common with all struggling with addiction. With this in mind, it is difficult to design clinical research that can replicate previous EEG studies of OUD so as not to repeat any common errors in research design and data analyses [102]. Additionally, our participants were recruited during various phases of withdrawal and active detoxification. This spectrum of symptom profiles may limit our results’ generalization to effects of ‘kindling’, or eventual limbic system hypersensitization seen in chronic alcohol detoxification and withdrawal [51,122]. Despite this potential limitation, our findings are in alignment with prior literature on ERP variance in this context, particularly similarly decreased P300 amplitudes and increased latencies observed in AUD and other chronic SUDs [82,103,123,124]. Cognitive changes associated with addictive or withdrawal states also mirror traditional treatment dogma encouraging those in early abstinence and/or withdrawal to avoid settings and company associated with prior drug-use, as sensory ‘triggers’ or ‘cues’ may disrupt CNS inhibition and promote relapse. Finally, self-reporting of how much or how long substance abuse has taken place can be a problem in SUD research; however, most SUD research must rely on such reports, and literature indicates that, for the most part, self-reporting is reliable in this type of investigation [103,125].

### 4.3. Correlation of ERPs with Wavelets

Wavelets were not correlated with stimulus condition as were ERPs. Further, ERPs and wavelets were uncorrelated overall, providing some evidence that they may index independent phenomena. Although the statistics presented in this paper are not sufficient to delineate precisely the cognitive and physiological processes that cause ERP and wavelet measurements, we can begin to form a new hypothesis for future research to test in SUDs and, in particular, for OUD. ERPs are well-established in their relationship to cognitive processes over decades of research, but the controversial nature of attributing causes to alpha or beta oscillations, in particular during the cognitive load of a feedback message in a Go/No-Go task, needs further experimental manipulations to prove or disprove causes. One possibility not presented in previous research is that there are many causes of alpha oscillations, and in the circumstances found in typical ERP studies, one or more of these causes can be present. Boredom, restlessness, or unfocused attention could contribute simultaneously, along with underlying personality traits and physiological properties that vary among participants. Routine physiological processes may be present as well, or even attenuated by each of the previously mentioned causes. Studies designed with alpha oscillations as a primary consideration could gather enough information about each of these variables to account for factors that vary the alpha oscillation effect. Regardless, oscillations during the feedback stimulus were robust, being evident on raw EEG waveforms, and were affected significantly by gender–OUD interactions. Studies are ongoing in our lab to determine if oscillations during the feedback message might be dependent upon dopamine levels in the brain.

### 4.4. Feedback Message Oscillation Studies

Although we did not expect to see evoked waveforms during the feedback stimulus presented at 500 ms after the subject’s response to the visual stimulus in this simple visual Go/No-Go task, upon inspection of grand-averaged waveforms in some subjects (Figure 3), it became evident that there was an appreciable evoked waveform that evinced differential response waveforms, similar to the waveforms induced by the visual stimulus that form the VEP and ERPs in the encompassing task. This observation led us to inspect the raw waveforms during the feedback message. It was obvious that a robust oscillation occurred therein for almost all subjects across all stimulus conditions and all around the head, but most evidently at the back of the head. Very rarely was a VEP or ERP evident on a single stimulus trial, as were the oscillations during the feedback message. As this observation was an unexpected outcome of the study, we did not generate hypotheses for this phenomenon, but report it here with our interpretation and its potential implications. Wavelet analysis of the oscillations evoked during the feedback stimulus was able to detect quantitative differences in delta, theta, alpha, and beta power during the feedback stimulus, with alpha and beta activity dominating the oscillation. However, given that the message itself was presented for only 500 ms, slower frequencies like delta and theta would not have had enough time to accurately sum in order to determine their power during this short epoch, hence their lower power. Surprisingly, unlike ERPs, alpha and beta activity did not vary significantly by stimulus condition in the simple Go/No-Go task. However, because of the variability in RT amongst participants and the presentation of the 500 ms feedback message (500 ms after the subject’s response to a Relevant stimulus), their amplitude varied considerably, except in those participants whose RTs for each trial were relatively less variable. As mentioned previously with ERPs (Figure 1 and Figure 2), because of temporal dispersion, differences in RT, grand-averaged waveforms, etc. often underestimate the significance of the effects due to temporal dispersion and other vagaries associated with averaging. This was especially true of the feedback message, given that it was dependent upon RT, at least for the Relevant stimulus. Thus, for this study, wavelet analysis was used instead of waveform grand-averaging to evaluate the power of alpha and beta oscillations during the feedback message. We found a significant difference between men and women for alpha and beta oscillations during the feedback message, and a significant difference between male OUD vs. non-OUD participants.

Alpha oscillations occur during visual tasks and occur mostly in the occipital and parietal lobes [126,127]. One study found perceptual stimuli in the visual field to be most relevant for accounting for power of alpha oscillations [128]. Background processing and visual perception activation have not been sufficient to explain alpha oscillations, so a cognitive explanation is a potentially fruitful cause to explore [129]. Although alpha oscillation often occurs in a resting participant, the alpha and beta oscillations in this study were robust and time-locked to the feedback message. There are several theories that compete in explaining why alpha oscillations occur in visual tasks and why they occur immediately preceding the presentation of a stimulus [130]. One theory is that they are tied to attention [131]. A competing theory is that they are tied to boredom [117,132]. Some researchers consider alpha oscillations as artifacts that can disrupt the collection of clean ERP components in event-focused ERP studies if care is not taken [102]. Alpha oscillations have also been attributed to participants’ anticipation of the upcoming stimulus [133,134]. As such, these brief but consistent alpha oscillations commonly occur immediately preceding a visual task in an ERP study. Researchers have sought to document the pattern of these alpha oscillations and what may cause them, and have targeted perceptual and attentional systems as likely causes of alpha oscillations [135]. The current study not only documents the presence of these types of alpha oscillations in all participants, but also provides evidence that they rival ERP components in their usefulness for discriminating their differences in the groups of participants and any interactions, including the gender-specific differences of brain functions in OUD. Many studies use short intervals between stimuli where ERPs are affected negatively by the alpha oscillations superimposed over those ERP components involved in the waveform [52,64,71,76,78,79,86,111,115,126,129,136,137,138,139,140,141,142,143,144,145,146,147,148,149]. Our study includes enough time between stimuli to avoid such overlap and evaluate oscillations with wavelets exclusively within the window of the feedback message. In this way, further studies can separate the effects of alpha oscillations and ERP components and test if they represent overlapping phenomena or if they are component-independent phenomena [117], possibly related to separate cognitive processes.

### 4.5. Future Studies

Across biological research over the past two decades, DA has consistently emerged as one of the greatest driving forces in substance abuse. As DA is associated with substance use and visual processing, we suggest that continuations of this study should explore whether DA levels vary across different visual tasks and EEG/ERP studies. Increasing sample sizes, especially when grouping by addiction status and sex, would be in line with these aims, as would incorporation of different stimuli. Another method of sampling for DA depletion using the same type of visual cognitive recognition task would also help generalize observed associations to other types of SUDs.

Given our results as well as previous findings on sex differences in object recognition tasks [118,119,150], we also suggest there is great need for further consideration of possible hormonal effects on DA neurotransmission in men and women, especially as these differences may relate to any form of addiction or other disinhibitory disorder [151]. This suggestion is in line with emerging clinical patterns. A robust study of substance abuse across 30,000 assessments completed in 220 different treatment centers between 2005 and 2008 indicated significant differences in men and women’s use of opioid pain pills [29]. Women out-used men overall, but not within opiate- or heroin-specific categories [152]. Women were also especially likely to abuse these substances when they were already problem drinkers. Better understanding the cognitive changes which may drive this gender-related susceptibility to different substances can potentially inform better practices for opioid prescriptions for pain management in women who may be more at risk for some types of substance abuse [81,125,153,154,155,156].

Furthermore, our findings may suggest that exploring simple, cost-effective visual processing tasks that could identify signs of relapse or risk for abuse is necessary and may be possible through observation of ERPs or VEPs. Previous research also demonstrates promise around the development of some type of light visualization [157,158] or other types of environmental enhancement stimuli that can improve inhibition systems such as those studied in cocaine addiction reversal [122,157,158,159]. Similar studies should be carried out for opioid addiction given recent estimates suggesting that societal effects of opioid misuse, abuse, and overdose deaths have surpassed those of cocaine.

Gazzaniga [160] notes that neuroscience is still in its infancy in understanding who and/or what is in charge of “free will” and what sort of neurobiological adaptations may or may not be within human control. In order to reduce societal stigmatization and enhance treatment outcomes, questions such as: “Why can’t they just stop?” must continue to be asked, and evidence-based answers to those harrowing inquiries must continue to be sought. Cognitive neuroscience and biological perspectives may serve as an especially firm foundation for understanding these aspects of human behavior.

Overall, as hypothesized, our results suggest that differences emerge in visual attention, cognitive processing, and physiology in men and women experiencing substance abuse and opiate addiction. Our results also suggest that the object recognition task used in the current study may be sensitive to such differences. Therefore, this task or other visual paradigms may further our understanding of biological mechanisms behind opiate addiction and its related inhibitory behaviors. We propose that such “behaviors may be [linked with] ‘out the gate’ immediate, evoked potentials from visual stimuli, [which] trigger some type of neurobiological cascade of neurotransmitters and component generation that can be ‘visualized’ on EEG studies” [160]. We hold that these uninhibited cascading neurotransmission events may lead to or represent signs of relapse in persons with OUD who are trying to stimulate the reward system with or without conscious awareness or deliberate attending to reward-associated stimuli in both women and men, but particularly in men. Our results offer further evidence that continued research in the area of DA neurotransmission and search for biomarkers in the visual system for opiate addiction and relapse as measured by EEG is warranted to illuminate reasons for sex-related differences in OUD prevalence and mortality rates [21,22].

## Figures and Tables

**Table 1:** Two-factor authentication of mixed-model multivariate analysis of variance (MANOVA) of the effects of opioid use disorder (OUD) nested within gender on P300 and N200 amplitudes and latencies and wavelet power levels.

A. Univariate Test						
	Gender Effects			OUD Effects within Gender		
	(Fixed)			(Random)		
Variable	*F*(1, 34)	*p*	*η* ^2^	*F*(2, 34)	*p*	*η* ^2^
P300						
Amplitude	25.16	<0.0001 ***	0.41	1	0.3788	0.033
Latency	3.52	0.0692	0.049	16.63	<0.0001 ***	0.462
N200						
Amplitude	14.5	0.0006 ***	0.289	0.94	0.4018	0.037
Latency	1.06	0.3105	0.019	9.84	0.0004 *	0.356
Wavelet Power						
Alpha	3.83	0.0585	0.095	1.21	0.3111	0.06
Beta	4.22	0.0477 ***	0.071	3.76	0.0333 *	0.165
B. Multivariate Tests						
	Gender Effects (Fixed)			Opioid Effects within Gender ‡ (Random)		
† Variable and Test	Value		*p*	Value		*p*
P300						
Wilks’ lambda	0.573515		0.0001 ***	0.469855		<0.0001 ***
Pillai’s trace	0.426485		0.0001 ***	0.554621		0.0002 ***
Hotelling-Lawley Trace	0.743632		0.0001 ***	1.076223		<0.0001 ***
Roy’s Greatest Root	0.743632		0.0001 ***	1.025422		<0.0001 ***
N200						
Wilks’ lambda	0.69395		0.0024 ***	0.598011		0.0018 ***
Pillai’s trace	0.30605		0.0024 ***	0.405014		0.0036 ***
Hotelling-Lawley Trace	0.441026		0.0024 ***	0.667152		0.0014 ***
Roy’s Greatest Root	0.441026		0.0024 ***	0.659482		0.0002 ***
Wavelet Power						
Wilks’ lambda	0.845376		0.0626	0.806848		0.1263
Pillai’s trace	0.154624		0.0626	0.193169		0.1355
Hotelling-Lawley Trace	0.182905		0.0626	0.239371		0.1206
Roy’s Greatest Root	0.182905		0.0626	0.239286		0.0261

†, ‡ Mixed-model correction does not affect the random factor. The mixed-model analysis is used to properly adjust the fixed factor with which the random factor is crossed or nested. Note * *p* < 0.05; *** *p* < 0.0001.

**Table 2:** Means and confidence intervals (CI) for the two-factor mixed-model MANOVA of the effects of gender and opioid use disorder (OUD) status nested within gender.

Variables	Means (95% CI)	Z-Score Means (95% CI)
P300 Amplitude		
Female		
OUD	2.257 (1.670, 2.844)	−0.358 (−0.698, −0.018)
Control	3.148 (2.201, 4.095)	0.158 (−0.390, 0.706)
Male		
OUD	5.082 (3.749, 6.415)	1.279 (0.507, 2.051)
Control	5.665 (4.514, 6.816)	1.616 (0.949, 2.283)
P300 Latency		
Female		
OUD	339.5 (309.8, 369.1)	−0.897 (−1.348, −0.446)
Control	460.7 (425.7, 495.8)	0.945 (0.413, 1.477)
Male		
OUD	470.4 (434.4, 506.4)	1.092 (0.545, 1.639)
Control	395.4 (359.2, 431.6)	−0.047 (−0.596, 0.502)
N200 Amplitude		
Female		
OUD	1.750 (1.459, 2.041)	−0.518 (−0.858, −0.178)
Control	1.710 (1.075, 2.345)	−0.553 (−1.101, −0.004)
Male		
OUD	2.620 (1.785, 3.455)	0.144 (−0.628, 0.917)
Control	3.240 (2.664, 3.816)	0.606 (−0.061, 1.273)
N200 Latency		
Female		
OUD	274.0 (252.7, 295.3)	−0.660 (−1.110, −0.209)
Control	304.7 (285.6, 323.8)	0.124 (−0.408, 0.656)
Male		
OUD	329.5 (305.1, 353.8)	0.755 (0.208, 1.302)
Control	271.0 (254.5, 287.5)	−0.737 (−1.286, −0.189)
Alpha Wavelet Power		
Female		
OUD	0.1107 (0.0651, 0.1563)	−0.795 (−1.278, −0.312)
Control	0.1498 (0.0918, 0.2078)	−0.381 (−0.995, 0.233)
Male		
OUD	0.1624 (0.1994, 0.2255)	−0.248 (−0.916, 0.420)
Control	0.2155 (0.1483, 0.2826)	0.314 (−0.397, 1.025)
Beta Wavelet Power		
Female		
OUD	0.1421 (0.1154, 0.1687)	−0.219 (−0.748, 0.310)
Control	0.1769 (0.1458, 0.2080)	0.472 (−0.146, 1.089)
Male		
OUD	0.1667 (0.1320, 0.2013)	0.269 (−0.418, 0.957)
Control	0.2175 (0.1863, 0.2487)	1.227 (0.659, 1.896)

**Table 3:** Correlation matrix for all event-related potentials (ERPs) and wavelets variables for the Relevant condition in the object recognition task.

	N200 Amplitude	P300 Amplitude	N200 Latency	P300 Latency	Alpha	Beta
N200 amplitude	1					
P300 amplitude	0.58	1				
N200 latency	0.04	0.04	1			
P300 latency	0.15	0.37	0.54	1		
alpha	0.03	0.01	0.27	0.03	1	
beta	0.15	−0.03	−0.17	−0.04	0.43	1

## Data Availability

The data supporting the findings of this study are available from the corresponding author upon reasonable request.
